# Novel role of cortactin in G protein-coupled receptor agonist-induced nuclear export and degradation of p21Cip1

**DOI:** 10.1038/srep28687

**Published:** 2016-07-01

**Authors:** Jagadeesh Janjanam, Gadiparthi N. Rao

**Affiliations:** 1 Department of Physiology, University of Tennessee Health Science Center, Memphis, TN 38163, USA

## Abstract

Monocyte chemotactic protein 1 (MCP1) stimulates phosphorylation of cortactin on Y421 and Y446 residues in a time-dependent manner and phosphorylation at Y446 but not Y421 residue is required for MCP1-induced CDK-interacting protein 1 (p21Cip1) nuclear export and degradation in facilitating human aortic smooth muscle cell (HASMC) proliferation. In addition, MCP1-induced cortactin tyrosine phosphorylation, p21Cip1 degradation and HASMC proliferation are dependent on Fyn activation. Upstream to Fyn, MCP1 stimulated C-C chemokine receptor type 2 (CCR2) and Gi/o and inhibition of either one of these molecules using their specific antagonists or inhibitors attenuated MCP1-induced cortactin tyrosine phosphorylation, p21Cip1 degradation and HASMC proliferation. Cortactin phosphorylation at Y446 residue is also required for another G protein-coupled receptor (GPCR) agonist, thrombin-induced p21Cip1 nuclear export and its degradation in promoting HASMC proliferation. Quite interestingly, the receptor tyrosine kinase (RTK) agonist, platelet-derived growth factor-BB (PDGF-BB)-induced p21Cip1 degradation and HASMC proliferation do not require cortactin tyrosine phosphorylation. Together, these findings demonstrate that tyrosine phosphorylation of cortactin at Y446 residue is selective for only GPCR but not RTK agonist-induced nuclear export and proteolytic degradation of p21Cip1 in HASMC proliferation.

Cell proliferation plays an essential role in the development of an organism and tissue repairing^1^. However, an increase in demand for cell proliferation due to chronic inflammatory responses, hormonal dysfunctions, compensation for tissue damage or disease leads to hyperplasia^2^. There are many commonly known clinical forms of hyperplasia among which intimal hyperplasia is the major cause of restenosis, characterized by arterial wall thickening with decreased arterial lumen space, which occurs as a response to vascular injury^3^. Vascular smooth muscle cell (VSMC) proliferation along with its migration into the tunica intima is the root cause of restenosis^4^,^5^. A variety of stimulants that are produced at the site of vascular injury appear to be involved in the pathogenesis of restenosis^4^. Among the many molecules identified, the artery produces a chemokine, monocyte chemotactic protein 1 (MCP1) acutely and robustly in response to injury^6^, which in turn, stimulates VSMC motility and multiplication leading to vascular wall remodeling^7^,^8^. Although many studies have reported a role for various signaling molecules in human aortic smooth muscle cell (HASMC) migration and proliferation, the role of cytoskeletal proteins in these effects are not well understood. In a recent study, we reported that cortactin, an actin binding protein, mediates MCP1-induced actin polymerization and HASMC migration^9^. Cortactin, which was initially identified as a Src substrate, was later found as a nucleation-promoting factor^10^,^11^ and its role in cell migration, endocytosis and vesicle trafficking has been well studied^12^.

Post-translational modifications of cortactin especially acetylation and phosphorylation were shown to govern its interactions with other cytoskeletal proteins in the modulation of cell migration^12^,^13^,^14^,^15^,^16^. Cortactin acetylation by histone acetyltransferase p300 neutralizes its charged lysine residues and inhibits its binding to F-actin leading to reduced cell migration^17^. On the other hand, cortactin deacetylation by histone deacetylases (HDACs) such as HDAC6 or HDAC8 and sirtuins such as sirtuin 1 (SIRT1) increases its binding to F-actin and promotes cell migration^17^,^18^,^19^. Cortactin phosphorylation at S405 and S418 by p21-activated kinase 1 (Pak1) and extracellular signal-regulated kinases 1/2 (ERK1/2) is required for its interaction with neural Wiskott-Aldrich syndrome protein (N-WASP) in promoting actin polymerization and lamellipodium formation^14^,^20^. Recently, we have reported that cortactin phosphorylation at S405 and S418 residues by protein kinase Cδ (PKCδ) is needed for its interaction with WASP family protein member 2 (WAVE2) in facilitating actin polymerization and VSMC migration^9^. In addition, cortactin was shown to be phosphorylated by several non-receptor tyrosine kinases such as the Src family of protein kinases, the Abelson (ABL) family of protein kinases, feline encephalitis virus-related (FER) kinase and spleen tyrosine kinase^14^,^16^,^21^,^22^. It was also reported that phosphorylation of mouse cortactin at Y421, Y466 and Y482 residues (equivalent to Y421, Y470 and Y486 residues in human cortactin) is required for its role in lamellipodia formation and cell migration^13^. Furthermore, human cortactin phosphorylation at Y446 residue has been reported to be required for its role in cellular protection from hyperosmotic stress-induced apoptosis^23^. Cortactin tyrosine phosphorylation has also been shown to play a role in endocytosis of various receptors^24^,^25^.

While the functional role of cortactin in cell migration and receptor endocytosis has been well studied, its role in cell proliferation is limited to a few studies. Overexpression of cortactin enhances serum- and epidermal growth factor-stimulated proliferation of head and neck squamous carcinoma cells^26^. In addition, it was shown that depletion of cortactin levels increases cyclin-dependent kinase inhibitors (CDKIs) leading to blockade of S-phase entry and cell cycle progression of 11q13-amplified head and neck squamous carcinoma cells^27^. A recent study has also shown that cortactin overexpression besides migration promotes SGC-7901 gastric cancer cell invasion and proliferation^28^. Cyclin-dependent kinase (CDK) inhibitors of the Cip/Kip family, namely, p21Cip1, p27Kip1, and p57Kip2 play an important role in the negative regulation of cell cycle progression^29^. Furthermore, it was reported that p21Cip1 besides inhibiting CDK2 activity also binds to, and suppresses proliferating cell nuclear antigen (PCNA) activity in blocking cell proliferation^30^. Based on all these reports it may be suggested that cortactin plays an important role in the modulation of cell proliferation. Despite these clues on the role of cortactin in cell proliferation, its involvement in VSMC proliferation and neointima formation is not known. Therefore, in the present study, we studied the role of cortactin in MCP1-induced HASMC proliferation. Here, we report that CCR2, Gi/o and Fyn-mediated phosphorylation of cortactin at Y446 residue is required for MCP1-induced p21Cip1 nuclear export and degradation leading to HASMC proliferation.

## Results

### Cortactin mediates MCP1-induced p21Cip1 degradation

**Figure 1:**
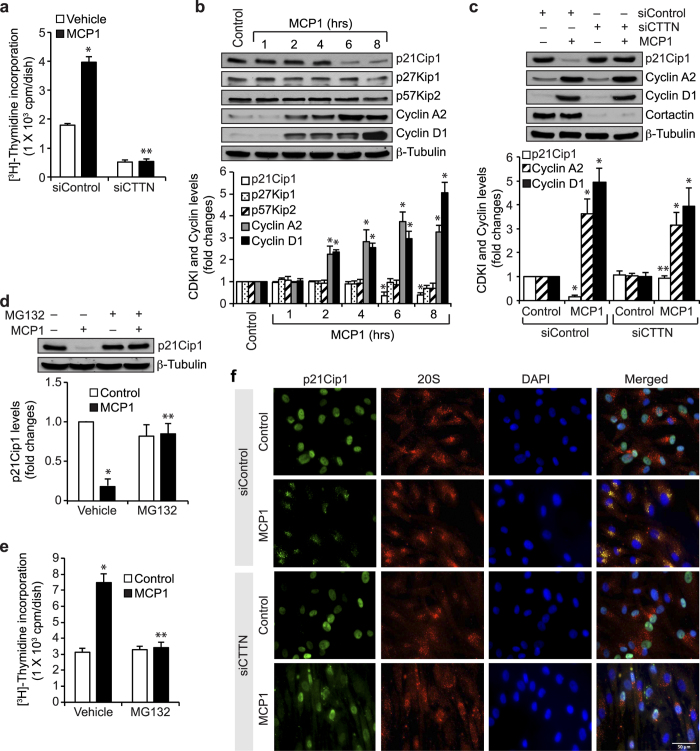
Cortactin mediates MCP1-induced p21Cip1 degradation and HASMC proliferation. (**a**) HASMCs that were transfected with siControl or siCortactin (100 nM) and growth-arrested were treated with vehicle or MCP1 (50 ng/ml) for 36 hrs and DNA synthesis was measured by [^3^H]-thymidine incorporation. (**b**) Equal amounts of protein from control and various time periods of MCP1-treated HASMCs were analyzed by Western blotting for the indicated CDKIs and cyclins using their specific antibodies and normalized to β-tubulin. (**c**) All the conditions were the same as in panel **a** except that after growth-arrest cells were treated with and without MCP1 for 8 hrs and cell extracts were prepared. Equal amounts of protein from control and each treatment were analyzed by Western blotting for p21Cip1, cylcin A2 and cyclin D1 levels. Equal amounts of protein from the same cell extracts were also analyzed by Western blotting for cortactin and β-tubulin levels to show the efficacy of the siRNA on its target and off target molecules levels. (**d**) Growth-arrested cells were treated with vehicle or MCP1 in the presence and absence of MG132 (10 μM) for 8 hrs, cell extracts were prepared and an equal amount of protein from control and each treatment was analyzed by Western blotting for p21Cip1 levels and normalized to β-tubulin. (**e**) All the conditions were the same as in panel **d** except that cells were treated with vehicle or MCP1 for 36 hrs and DNA synthesis was measured. (**f**) All the conditions were the same as in panel **a** except that cells were treated with vehicle or MCP1 for 6 hrs and immunostained for p21Cip1 and 20Sα/β using their specific antibodies. The bar graphs in panels **a–e** represent Mean ± SD values of three independent experiments. *p < 0.05 vs vehicle control or siControl; **p < 0.05 vs MCP1 or siControl + MCP1.

Studies from various laboratories including ours have demonstrated that cortactin plays an important role in cell migration^9^,^15^,^31^. Although cortactin role in cell migration has been well established, its role in cell proliferation is not clear. Since MCP1 acts as a chemoattractant and mitogen to VSMCs^7^,^32^, we asked the question whether cortactin plays a role in MCP1-induced HASMC proliferation. To address this, we applied a small interfering RNA (siRNA) approach to deplete cortactin levels and studied its role in MCP1-induced HASMC proliferation. Depletion of cortactin levels by its siRNA molecules prevented MCP1-induced HASMC DNA synthesis (Fig. 1a). In order to understand the mechanism(s) by which cortactin plays a role in MCP1-induced HASMC DNA synthesis, first we have studied the effect of MCP1 on cell cycle regulatory proteins such as cyclins (cyclin D1 and cyclin A2) and CDKIs (p21Cip1, p27Kip1 and p57Kip2). MCP1 while having no effect on p27Kip1 and p57Kip2 levels induced cyclin D1 and cyclin A2 expression and downregulated p21Cip1 levels in a time-dependent manner (Fig. 1b). In addition, siRNA-mediated depletion of cortactin levels without affecting cyclin D1 and cyclin A2 levels inhibited MCP1-induced p21Cip1 downregulation (Fig. 1c). Many studies have reported that p21Cip1 undergoes proteasome-mediated degradation in response to mitogens^33^,^34^. In order to understand the mechanisms by which MCP1 induces p21Cip1 downregulation, we studied the effect of MG132, a potent inhibitor of proteasomes^34^. MG132 blocked MCP1-induced p21Cip1 downregulation and HASMC DNA synthesis indicating that p21Cip1 undergoes proteasome-mediated degradation, thereby facilitating HASMC proliferation in response to MCP1 (Fig. 1d,e). To obtain evidence supporting this point, we have studied the effect of MCP1 on p21Cip1 and 20S proteasome co-localization. As measured by co-immunostaining for p21Cip1 and proteasome 20Sα/β subunits, MCP1 induced p21Cip1 co-localization with 20S proteasomes in a time-dependent manner (Supplementary Fig. S1). In addition, siRNA-mediated depletion of cortactin levels inhibited MCP1-induced p21Cip1 co-localization with 20S proteasomes (Fig. 1f).

### Tyrosine phosphorylation of cortactin is required for MCP1-induced p21Cip1 nuclear export and degradation

**Figure 2:**
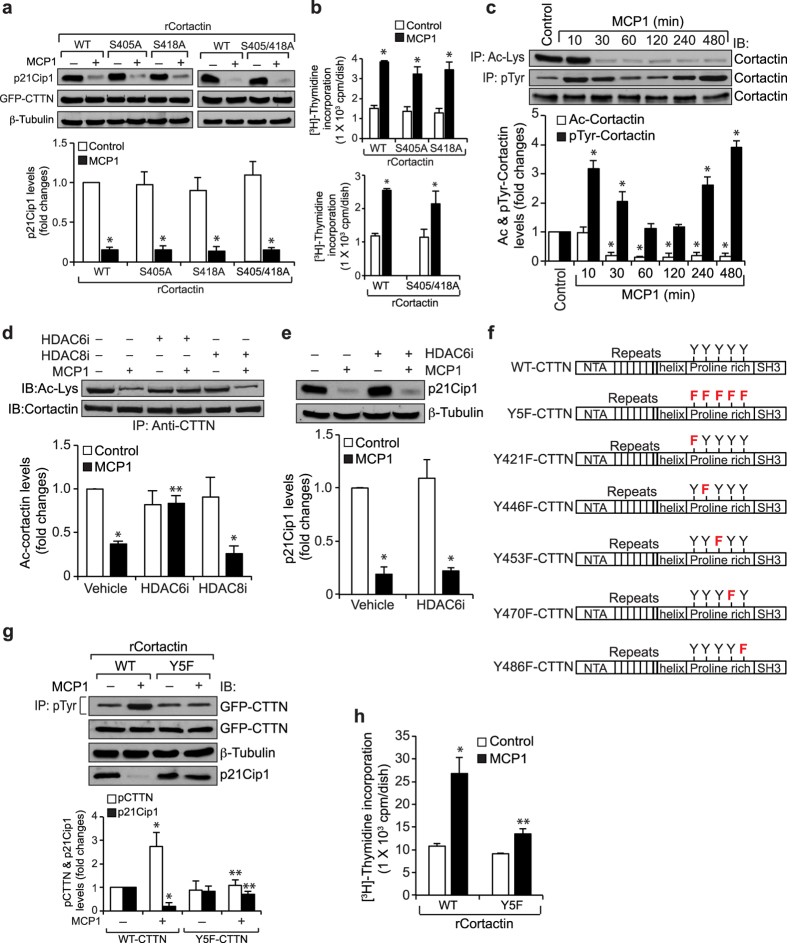
Cortactin tyrosine phosphorylation is required for MCP1-induced p21Cip1 degradation and HASMC proliferation. (**a**) HASMCs that were transfected with WT, S405A, S418A or S405/418A mutant cortactin expression vector and treated with vehicle or MCP1 for 8 hrs were analyzed by immunoblotting for p21Cip1 levels and the blot was reprobed for GFP or β-tubulin to show the overexpression of WT or mutant cortactin or normalization. (**b**) All the conditions were the same as in panel **a** except that cells were subjected to MCP1-induced DNA synthesis. (**c**) Equal amounts of protein from control and the indicated treatments were immunoprecipitated with anti-acetyl lysine or anti-phosphotyrosine antibodies and the immunocomplexes were analyzed by immunoblotting for cortactin. The same cell extracts were also analyzed by immunoblotting for cortactin. (**d**) HASMCs were treated with vehicle or MCP1 in the presence and absence of HDAC6i or HDAC8i (10 μM) for 6 hrs and equal amounts of protein from control and each treatment were immunoprecipitated with anti-cortactin antibodies and the immunocomplexes were analyzed by immunoblotting with anti-acetyl lysine antibody and normalized to cortactin. (**e**) HASMCs were treated with vehicle or MCP1 in the presence and absence of HDAC6 inhibitor for 8 hrs and equal amounts of protein from control and each treatment were analyzed by immunoblotting for p21Cip1 and normalized to β-tubulin. (**f**) The cloning strategy for cortactin expression vectors is presented and the mutant amino acid is shown in red. (**g**) HASMCs were transfected with WT or Y5F mutant cortactin expression vector, treated with vehicle or MCP1 for 8 hrs and equal amounts of protein from control and each treatment were immunoprecipitated with anti-pTyr antibody and the immunocomplexes were analyzed by immunoblotting for GFP. Same cell extracts were also analyzed by immunoblotting for GFP-CTTN and p21Cip1 and normalized to β-tubulin. (**h**) All the conditions were the same as in panel **g** except that cells were subjected to MCP1-induced DNA synthesis. The bar graphs in (**a**–**e**,**g**,**h**) represent Mean ± SD values of three independent experiments. *p < 0.05 vs vehicle control or WT-CTTN; **p < 0.05 vs MCP1 or WT-CTTN + MCP1. HDAC6i, HDAC6 inhibitor; HDAC8i, HDAC8 inhibitor.

Recently, we have reported that MCP1 induces serine phosphorylation of cortactin on S405 and S418 residues in enhancing F-actin stress fiber formation and HASMC migration^9^. Therefore, to understand the mechanisms by which cortactin mediates MCP1-induced p21Cip1 nuclear export and degradation, we have examined for the role of its serine phosphorylation. Forced expression of S405A or S418A cortactin single or double mutants had no effect on MCP1-induced p21Cip1 degradation and HASMC DNA synthesis (Fig. 2a,b). Next, we have explored the role of its acetylation and tyrosine phosphorylation in MCP1-induced p21Cip1 degradation. MCP1 induced cortactin deacetylation and its tyrosine phosphorylation in a time-dependent manner (Fig. 2c). It was previously reported that HDAC6 and HDAC8 mediate cortactin deacetylation^17^,^19^. Based on this information, we have tested the role of HDAC6 and HDAC8 in MCP1-induced cortactin deacetylation. Inhibition of HDAC6 but not HDAC8 blocked MCP1-induced cortactin deacetylation (Fig. 2d). However, inhibition of HDAC6 had no effect on MCP1-induced p21Cip1 degradation (Fig. 2e), indicating a lack of a role of cortactin deacetylation in MCP1-induced p21Cip1 degradation. Next, we examined for the role of cortactin tyrosine phosphorylation. Since there are five potential tyrosine residues in human cortactin, initially we have mutated all of these five tyrosine (Y421, Y446, Y453, Y470 and Y486) residues in GFP-cortactin expression vector using multi site-directed mutagenesis and named the resulting expression vector as Y5F-CTTN (Fig. 2f). Overexpression of Y5F-CTTN mutant blocked MCP1-induced cortactin tyrosine phosphorylation, p21Cip1 degradation and HASMC DNA synthesis, suggesting that cortactin tyrosine phosphorylation plays a role in MCP1-induced p21Cip1 depletion and HASMC proliferation (Fig. 2g,h).

**Figure 3:**
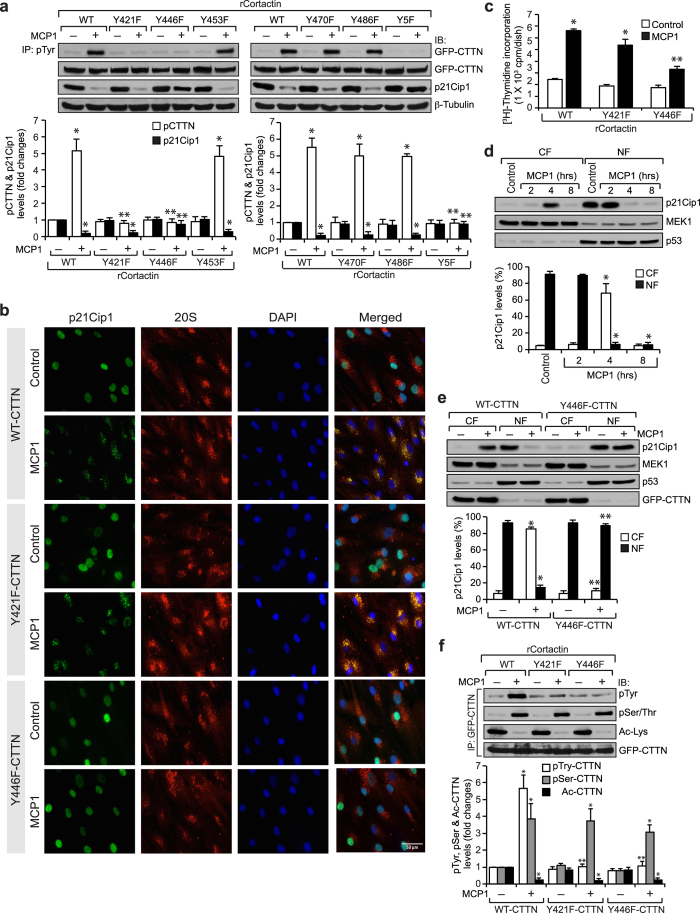
MCP1-induced p21Cip1 degradation requires cortactin phosphorylation at Y446. (**a**) Cells transfected with the indicated cortactin expression vectors were growth-arrested, treated with vehicle or MCP1 for 6 hrs and equal amount of protein from control and each treatment were immunoprecipitated with anti-pTyr antibodies and the immunocomplexes were analyzed by immunoblotting for GFP-CTTN. Same cell extracts were also analyzed by immunoblotting for GFP-CTTN and p21Cip1 levels and normalized to β-tubulin. (**b**) Cells were transfected with Myc-tagged WT or mutant (Y421F or Y446F) cortactin expression vectors, growth-arrested, treated with vehicle or MCP1 for 6 hrs and co-immunostained for p21Cip1 and proteasome 20Sα/β using their specific antibodies. (**c**) All the conditions were same as in panel **b** except that cells were subjected to MCP1-induced DNA synthesis. (**d**) The cytoplasmic and nuclear fractions (CF and NF, respectively) of control and the indicated time periods of MCP1-treated HASMCs were analyzed by Western blotting for p21Cip1 levels and reprobed for MEK1 and p53 levels to show the relative purity of the cytoplasmic and nuclear fractions, respectively. (**e**) Cells were transfected with GFP-tagged WT or Y446F mutant cortactin expression vector, growth-arrested, treated with vehicle or MCP1 for 4 hrs, cell extracts prepared and an equal amount of protein form the cytoplasmic and nuclear fractions was analyzed by Western blotting for p21Cip1, MEK1 and p53 levels as described in panel **d**. The same blot was reprobed for GFP to show the overexpression of WT or mutant cortactin levels. (**f**) Cells were transfected with GFP-tagged WT or mutant (Y421F or Y446F) cortactin expression vectors, growth-arrested, treated with vehicle or MCP1 for 6 hrs and equal amounts of protein from each condition were immunoprecipitated with anti-GFP antibodies and the immunocomplexes were analyzed by immunoblotting using anti-pTyr, anti-pSer/Thr or anti-Ac-Lys antibodies and normalized to GFP-CTTN. The bar graphs in panels **a** and **c–f** represent Mean ± SD values of three independent experiments. *p < 0.05 vs vehicle control or WT-CTTN; **p < 0.05 vs MCP1 or WT-CTTN + MCP1.

Since cortactin tyrosine phosphorylation is required for MCP1-induced p21Cip1 degradation and HASMC proliferation, we next wanted to identify the tyrosine residue(s) that are phosphorylated by MCP1. To gain insights into this, we mutated each of the five tyrosine residues to phenylalanine by site-directed mutagenesis and tested their effects on MCP1-induced cortactin tyrosine phosphorylation. While Y453F, Y470F, and Y486F cortactin mutants had no effect, the Y421F and Y446F cortactin mutants prevented MCP1-induced cortactin tyrosine phosphorylation, suggesting that MCP1 induces phosphorylation of cortactin at Y421 and Y446 residues (Fig. 3a). However, only Y446F but not the Y421F cortactin mutant abolished MCP1-induced p21Cip1 degradation (Fig. 3a). In addition, Y446F but not the Y421F cortactin mutant inhibited p21Cip1 and 20S proteasome co-localization as well as HASMC DNA synthesis induced by MCP1, suggesting that cortactin phosphorylation at Y446 residue is required for MCP1-induced p21Cip1 nuclear export and its degradation leading to HASMC proliferation (Fig. 3b,c). To confirm p21Cip1 and 20S proteasome co-localization, we have used a different antibody that recognizes proteasome 20Sα1, 2, 3, 5, 6 & 7 subunits for coimmunostaining along with p21Cip1 and obtained similar results as those shown in Fig. 3b (Supplementary Fig. S2). We further tested the effect of cortactin Y446F mutant on the translocation of p21Cip1 from the nucleus to the cytoplasm. MCP1 induced translocation of p21Cip1 from the nucleus to the cytoplasm in a time-dependent manner, appearing only in the cytoplasm at 4 hrs and disappearing both in the nucleus and the cytoplasm at 8 hrs, indicating its degradation at the latter time point (Fig. 3d) and overexpression of cortactin Y446F mutant prevented these effects (Fig. 3e). Since forced expression of cortactin Y446F mutant blocked MCP1-induced p21Cip1 translocation from the nucleus to the cytoplasm, it is likely that this mutant is acting as a dominant negative mutant. To show the transfection efficiency and differential effects of WT and Y446F cortactin mutant on MCP1-induced p21Cip1 nuclear export and degradation visually, cells were transfected with GFP-WT or GFP-Y446F cortactin mutant, growth-arrested, treated with and without MCP1 in the presence and absence of MG132 for 6 hrs and co-immunostained for GFP along with p21Cip1. First, since most of the cells exhibited green fluorescence, it is likely that almost all the cells were transfected with GFP-WT or GFP-Y446F cortactin mutant (Supplementary Fig. S3a). Second, in cells expressing WT cortactin, the p21Cip1 was found only in the nucleus and in response to treatment with MCP1, its presence was barely detected only in the cytoplasm indicating its degradation. In addition, in the presence of MG132, the p21Cip1 was found to be accumulated mostly in the cytoplasm of the MCP1-treated cells, suggesting that inhibition of proteasomal activity only prevented MCP1-induced p21Cip1 degradation but not its translocation from the nucleus to the cytoplasm (Supplementary Fig. S3b). On the other hand, in cells transfected with Y446F cortactin mutant, the p21Cip1 was retained only in the nucleus regardless of the treatment with and without MCP1 in the presence or absence of MG132 (Supplementary Fig. S3b). These findings suggest that cortactin tyrosine phosphorylation at Y446 residue plays a role in p21Cip1 translocation from the nucleus to the cytoplasm. To find whether tyrosine phosphorylation of cortactin leads to its serine phosphorylation or deacetylation in mediating MCP1-induced p21Cip1 nuclear export and degradation, we tested the effects of Y421F and Y446F cortactin mutants in MCP1-induced cortactin serine phosphorylation and its deacetylation. Neither mutant had any effect on MCP1-induced cortactin serine phosphorylation or its deacetylation (Fig. 3f).

### Fyn mediates MCP1-induced cortactin tyrosine phosphorylation, p21Cip1 degradation and HASMC proliferation

**Figure 4:**
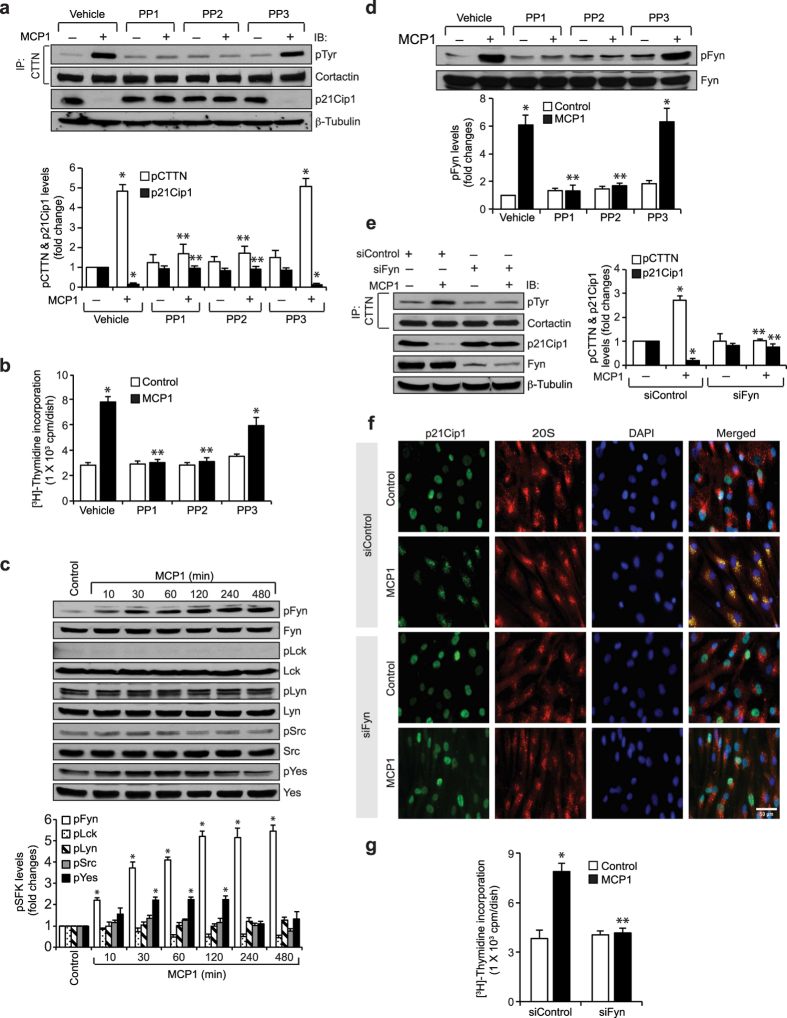
Fyn mediates MCP1-induced cortactin tyrosine phosphorylation, p21Cip1 degradation and HASMC proliferation. (**a**) Growth-arrested cells were treated with vehicle or MCP1 in the presence and absence of PP1 (10 μM), PP2 (10 μM), or PP3 (10 μM) for 6 hrs and the cell extracts were prepared. An equal amount of protein from control and each treatment was immunoprecipitated with anti-cortactin antibodies and the immunocomplexes were analyzed by immunoblotting with anti-pTyr antibodies and normalized for cortactin. Equal amounts of protein from the same cell extracts were also analyzed by Western blotting for p21Cip1 levels and normalized to β-tubulin. (**b**) All the conditions were same as in panel **a** except that cells were subjected to MCP1-induced DNA synthesis. (**c**) Equal amounts of protein from control and various time periods of MCP1-treated HASMCs were analyzed by Western blotting for phosphorylation levels of the indicated SFKs using their phosphospecific antibodies and normalized to their total levels. (**d**) All the conditions were the same as panel **a** except that an equal amount of protein from control and each treatment was analyzed by Western blotting for pFyn levels and normalized to total Fyn levels. (**e**) HASMCs that were transfected with siControl or siFyn and growth-arrested were treated with vehicle or MCP1 for 6 hrs, cell extracts prepared and an equal amount of protein from control and each treatment was immunoprecipitated with anti-cortactin antibodies and the immunocomplexes were analyzed by Western blotting with anti-pTyr antibodies and normalized for cortactin. Equal amounts of protein from the same cell extracts were also analyzed by Western blotting for p21Cip1 or Fyn levels and normalized to β-tubulin. (**f**) All the conditions were same as in panel **e** except that cells were treated with vehicle or MCP1 for 6 hrs and co-immunostained for p21Cip1 and 20Sα/β. (**g**) All the conditions were the same as in panel **e** except that cells were subjected to MCP1-induced DNA synthesis. The bar graphs in panels **a–e** and **g** represent Mean ± SD values of three independent experiments. *p < 0.05 vs vehicle control or siControl; **p < 0.05 vs MCP1 or siControl + MCP1.

It was reported that cortactin is a substrate for the Src family of the kinases (SFKs)^12^,^35^. Therefore, to explore the mechanisms of cortactin tyrosine phosphorylation, we tested the effects of PP1 and PP2, two potent SFK inhibitors, and their structural analog PP3, which does not affect SFK, on MCP1-induced cortactin tyrosine phosphorylation, p21Cip1 degradation and HASMC DNA synthesis. PP1 and PP2 but not PP3 blocked MCP1-induced cortactin tyrosine phosphorylation, p21Cip1 degradation, p21Cip1 co-localization with 20S proteasomes and HASMC DNA synthesis (Fig. 4a,b and Supplementary Fig. S4), suggesting that SFKs mediate MCP1-induced cortactin tyrosine phosphorylation, thereby causing p21Cip1 nuclear export and degradation leading to HASMC proliferation. In order to identify the specific SFK mediating cortactin tyrosine phosphorylation, we have studied the time course effect of MCP1 on phosphorylation of Fyn, Lck, Lyn, Src and Yes. Among the SFKs tested, only Fyn was found to be phosphorylated by MCP1 in a time-dependent manner (Fig. 4c) and this effect was blocked by both PP1 and PP2 (Fig. 4d). Based on these observations, we next tested the role of Fyn in MCP1-induced cortactin tyrosine phosphorylation, p21Cip1 degradation, p21Cip1 and 20S proteasome co-localization and HASMC DNA synthesis. Downregulation of Fyn levels by its siRNA molecules blocked MCP1-induced cortactin tyrosine phosphorylation and p21Cip1 degradation (Fig. 4e). Consistent with these observations, depletion of Fyn levels also prevented p21Cip1 co-localization with 20S proteasomes and inhibited HASMC DNA synthesis (Fig. 4f,g).

### Gi/o mediates MCP1-induced Fyn and cortactin tyrosine phosphorylation, p21Cip1 degradation and HASMC proliferation

**Figure 5:**
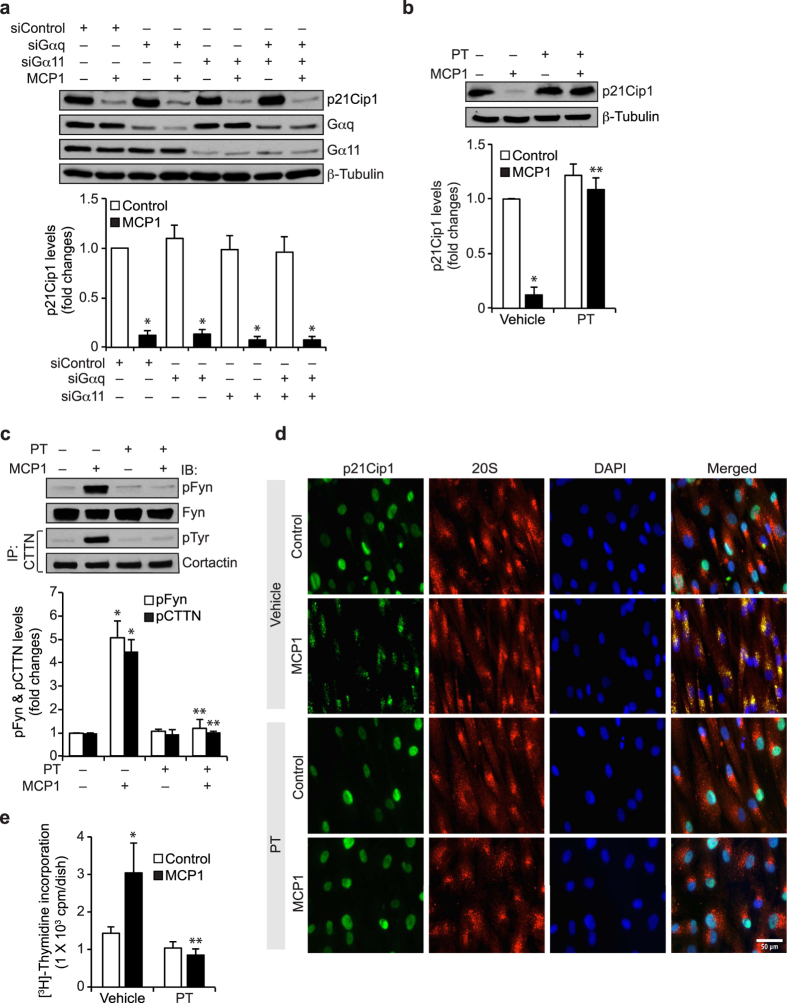
Gi/o-mediates MCP1-induced Fyn and cortactin tyrosine phosphorylation, p21Cip1 degradation and HASMC proliferation. (**a**) HASMCs that were transfected with siControl, siGαq or siGα11 and growth-arrested were treated with vehicle or MCP1 for 8 hrs and cell extracts were prepared. Equal amounts of protein from control and each treatment were analyzed by Western blotting for p21Cip1 levels and normalized to β-tubulin. Equal amounts of protein from the same cell extracts were also analyzed by Western blotting for Gαq and Gα11 levels to show the efficacy of the siRNA on the target molecule level. (**b**) Growth-arrested cells were treated with vehicle or MCP1 in the presence and absence of PT (50 ng/ml) for 8 hrs and the cell extracts were prepared. Equal amounts of protein from control and each treatment were analyzed by Western blotting for p21Cip1 levels and normalized to β-tubulin. (**c**) All the conditions were same as in panel **b** except that equal amounts of protein from control and each treatment were analyzed by Western blotting for pFyn levels using anti-pFyn antibodies and normalized to Fyn. Equal amounts of protein from the same cell extracts were also immunoprecipitated with anti-cortactin antibodies and the immunocomplexes were analyzed by Western blotting with anti-pTyr antibodies and normalized to cortactin. (**d**) All the conditions were the same as in panel **b** except that cells were treated with vehicle or MCP1 for 6 hrs and co-immunostained for p21Cip1 and 20Sα/β using their specific antibodies. (**e**) All the conditions were the same as in panel **b** except that cells were subjected to MCP1-induced DNA synthesis. The bar graphs in panels **a,b,d** and **e** represent Mean ± SD values of three independent experiments. *p < 0.05 vs vehicle control or siControl; **p < 0.05 vs MCP1 or siControl + MCP1.

In our previous study we have demonstrated that Gαq/11 mediates MCP1-induced cortactin serine phosphorylation via PLCβ3-dependent PKCδ activation^9^. Therefore, we tested the role of Gαq/11 in MCP1-induced p21Cip1 degradation. However, depleting Gαq/11 levels had no effect on MCP1-induced p21Cip1 degradation (Fig. 5a), suggesting a lack of a role for Gαq/11 in MCP1-induced p21Cip1 downregulation. Based on these observations, we next tested the effect of pertussis toxin (PT), specific inhibitor of Gαi/o, on MCP1-induced p21Cip1 degradation and found that it inhibits MCP1-induced p21Cip1 degradation (Fig. 5b). In addition, PT blocked MCP1-induced Fyn and cortactin tyrosine phosphorylation, p21Cip1 and 20S proteasome co-localization and HASMC DNA synthesis (Fig. 5c–e). These results suggest that Gαi/o mediates MCP1-induced Fyn-dependent cortactin tyrosine phosphorylation, p21Cip1 degradation and HASMC proliferation.

### CCR2 but not CCR4 activation is required for Gi/o-dependent Fyn and cortactin tyrosine phosphorylation in MCP1-induced p21Cip1 degradation and HASMC proliferation

**Figure 6:**
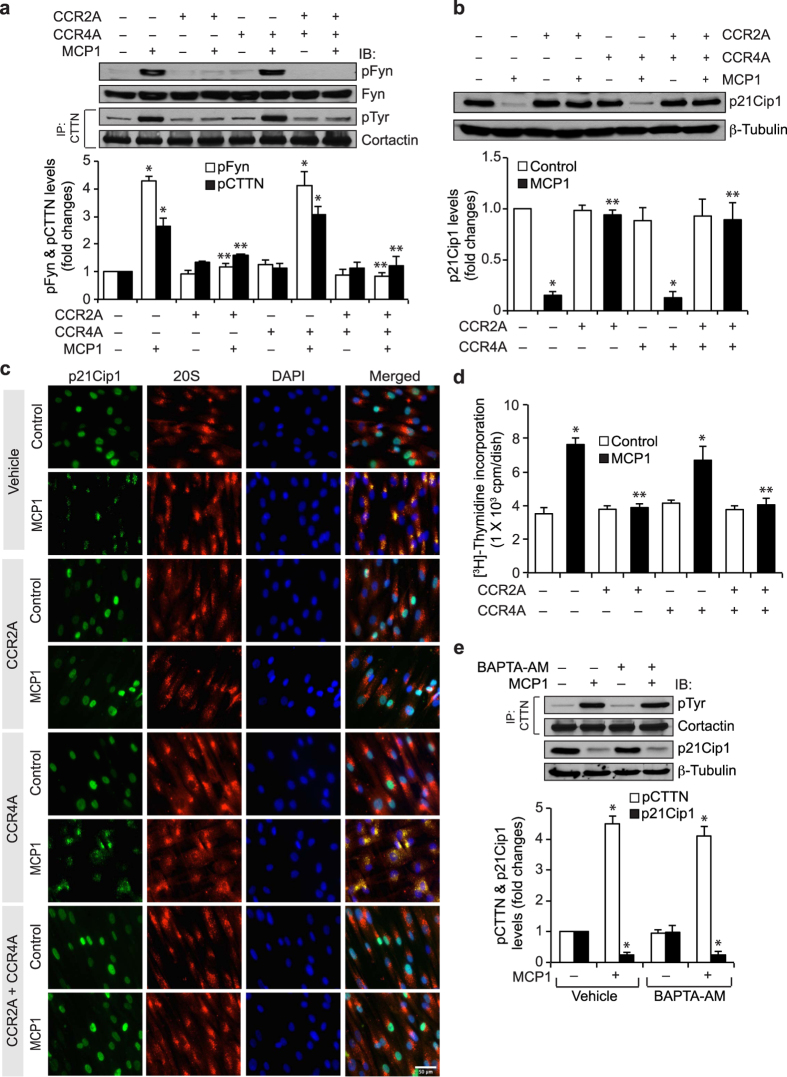
CCR2 but not CCR4 activation is required for cortactin-mediated MCP1-induced p21Cip1 degradation. (**a**) Growth-arrested HASMCs were treated with vehicle or MCP1 in the presence and absence of CCR2 antagonist (CCR2A) or CCR4 antagonist (CCR4A) for 6 hrs and the cell extracts were prepared. Equal amounts of protein from control and each treatment were analyzed by Western blotting for pFyn levels using anti-pFyn antibodies and normalized to Fyn. Equal amounts of protein from the same cell extracts were also immunoprecipitated with anti-cortactin antibodies and the immunocomplexes were analyzed by Western blotting with anti-pTyr antibodies and normalized to cortactin. (**b**) All the conditions were the same as in panel **a** except that equal amounts of protein from control and each treatment were analyzed by Western blotting for p21Cip1 levels using its specific antibodies and normalized to β-tubulin. (**c**) All the conditions were the same as in panel **a** except that cells were treated with vehicle or MCP1 for 6 hrs and co-immunostained for p21Cip1 and 20Sα/β using their specific antibodies. (**d**) All the conditions were the same as in panel **a** except that cells were subjected to MCP1-induced DNA synthesis. (**e**) Growth-arrested cells were treated with vehicle or MCP1 in the presence and absence of BAPTA-AM (10 μM) for 6 hrs and cell extracts were prepared. An equal amount of protein from control and each treatment was immunoprecipitated with anti-cortactin antibodies and the immunocomplexes were analyzed by immunoblotting with anti-pTyr antibodies and normalized for cortactin. Equal amounts of protein from the same cell extracts were also analyzed by Western blotting for p21Cip1 levels and normalized to β-tubulin. The bar graphs in panels **a,b,d** and **e** represent Mean ± SD values of three independent experiments. *p < 0.05 vs vehicle control; **p < 0.05 vs MCP1.

MCP1 mediates its effects via its receptors CCR2 and CCR4^36^. However, in HASMCs, it is not known which of these receptors mediate MCP1-induced Fyn and cortactin tyrosine phosphorylation, p21Cip1 degradation and HASMC proliferation. We have recently reported that both CCR2 and CCR4 are present in HASMCs^9^. Therefore, to study the role of these receptors in MCP1-induced HASMC proliferation, we used their antagonists, CCR2A and CCR4A, respectively. CCR2A but not CCR4A inhibited MCP1-induced Fyn and cortactin tyrosine phosphorylation, p21Cip1 degradation, p21Cip1 and 20S proteasome co-localization and HASMC DNA synthesis (Fig. 6a–d). These findings indicate that CCR2 is the major receptor in mediating MCP1-induced p21Cip1 degradation and HASMC proliferation. It was reported that thrombin, a GPCR agonist, activates SFKs in calcium-dependent manner^37^. Since MCP1-induced cortactin tyrosine phosphorylation and p21Cip1 degradation are dependent on Fyn activation, we have tested the role of calcium. BAPTA-AM, a cell-permeable calcium chelator, had no effect on MCP1-induced cortactin tyrosine phosphorylation or p21Cip1 degradation (Fig. 6e). These findings show that MCP1 induces cortactin tyrosine phosphorylation and p21Cip1 degradation in a manner that is independent of calcium mobilization.

### Cortactin tyrosine phosphorylation is selective for GPCR but not RTK agonist-induced p21Cip1 degradation and HASMC proliferation

**Figure 7:**
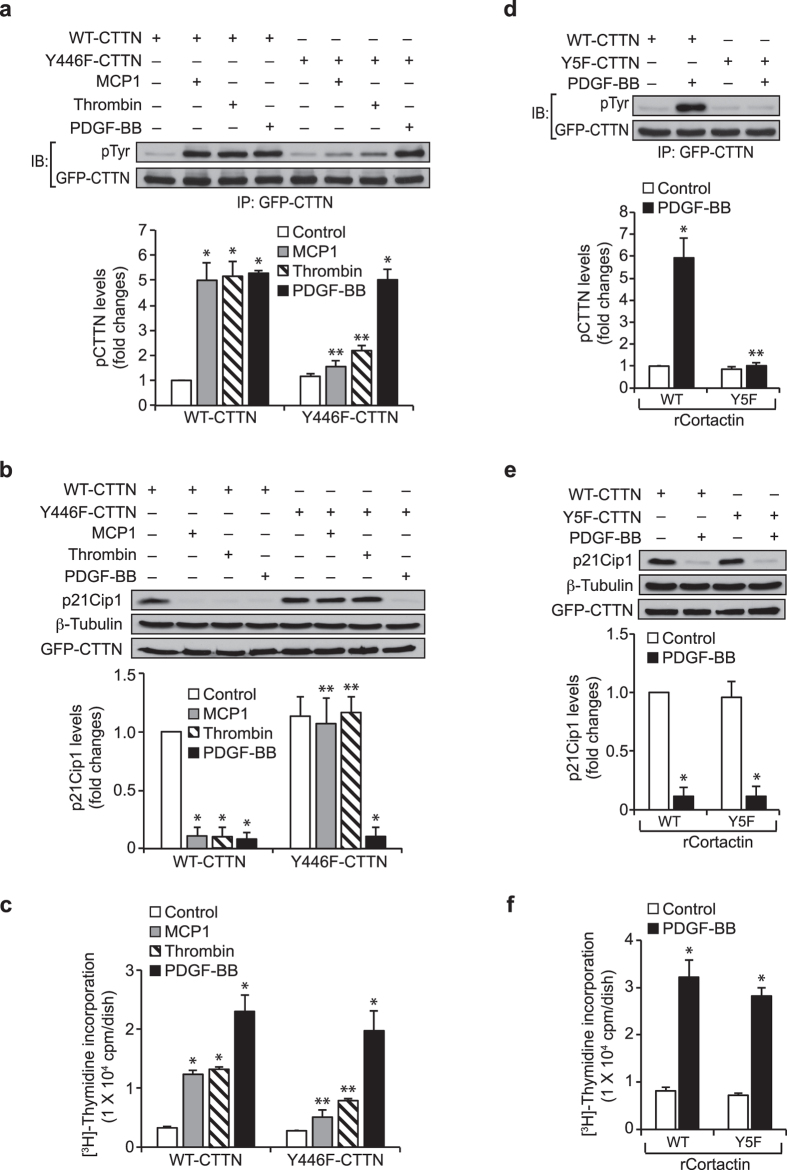
Cortactin phosphorylation at Y446 residue is required for only GPCR agonist but not RTK agonist-induced p21Cip1 degradation and HASMC proliferation. (**a**) HASMCs were transfected with GFP-tagged WT or Y446F mutant cortactin expression vector, growth-arrested, treated with vehicle, MCP1 (50 ng/ml), thrombin (0.5 U/ml) or PDGF-BB (20 ng/ml) for 6 hrs and cell extracts were prepared. Equal amounts of protein from control and each treatment were immunoprecipitated with anti-GFP antibodies and the immunocomplexes were analyzed by Western blotting with anti-pTyr antibodies and normalized for GFP. (**b**) All the conditions were the same as panel **a** except that cells were treated with vehicle or the indicated agonist for 8 hrs and equal amounts of protein from control and each treatment were analyzed by Western blotting for p21Cip1 levels and normalized to β-tubulin. (**c**) All the conditions were same as in panel **a** except that cells were subjected to the indicated agonist-induced DNA synthesis. (**d**) Cells were transfected with WT or Y5F mutant cortactin expression vector, growth-arrested, treated with vehicle or PDGF-BB for 6 hrs and cell extracts were prepared. Equal amount of protein from control and each treatment were immunoprecipitated with anti-GFP antibodies, and the immunocomplexes were analyzed by Western blotting with anti-pTyr antibodies and normalized to GFP. (**e**) All the conditions were the same as in panel **d** except that cells were treated with vehicle or PDGF-BB for 8 hrs and equal amounts of protein from each condition were analyzed by Western blotting for p21Cip1 levels using its specific antibodies and normalized to β-tubulin. The same cell extracts were also analyzed by Western blotting for GFP to show the overexpression of WT or mutant cortactin. (**f**) All the conditions were the same as in panel **d** except that cells were subjected to PDGF-BB-induced DNA synthesis. The bar graphs in panels **a–f** represent Mean ± SD values of three independent experiments. *p < 0.05 vs WT-CTTN; **p < 0.05 vs WT-CTTN+ the indicated agonist.

To find the specificity of cortactin tyrosine phosphorylation signaling in MCP1-induced HASMC proliferation, we first tested the effect of Y446F cortactin mutant on MCP1, thrombin and PDGF-BB-induced cortactin tyrosine phosphorylation. Surprisingly, overexpression of Y446F cortactin mutant prevented only MCP1 and thrombin but not PDGF-BB-induced cortactin tyrosine phosphorylation (Fig. 7a). These findings infer that only GPCR agonists but not RTK agonists induce cortactin phosphorylation at Y446 residue. To find whether cortactin phosphorylation at Y446 residue is also required for only GPCR agonist-induced p21Cip1 degradation and HASMC proliferation, we next tested the effect of Y446F cortactin mutant on MCP1, thrombin and PDGF-BB-induced p21Cip1 downregulation and HASMC DNA synthesis. Consistent with its effect on cortactin tyrosine phosphorylation, Y446F cortactin mutant inhibited only MCP1 and thrombin but not PDGF-BB-induced p21Cip1 degradation and HASMC DNA synthesis (Fig. 7b,c). In order to examine whether cortactin phosphorylation at other tyrosine residues might be involved in PDGF-BB-induced p21Cip1 degradation and HASMC proliferation, we studied the effect of Y5F cortactin mutant. Interestingly, Y5F cortactin mutant while blocking cortactin tyrosine phosphorylation had no effect on PDGF-BB-induced p21Cip1 downregulation and HASMC DNA synthesis (Fig. 7d–f).

## Discussion

Many studies have demonstrated a role for cortactin in the regulation of actin cytoskeleton remodeling and cell migration^11^,^12^. Cortactin due to its up regulation during cancer invasion has also been considered as an important biomarker for melanoma, colorectal cancer and glioblastoma^38^,^39^. In addition, cortactin has been reported to play a role in endocytosis, invadopodia and podosome formation^40^,^41^. The functional role of cortactin is mainly regulated by its post-translational modifications such as acetylation and phosphorylation^12^. Previous studies have shown that deacetylation of cortactin by HDACs or sirtuins increases its binding capacity to F-actin and promotes cell migration^17^,^18^,^19^. On the other hand, phosphorylation of cortactin at S405 and S418 residues has been shown to promote G-actin polymerization and lamellipodium formation^14^,^20^. In addition, it was demonstrated that cortactin via its tyrosine phosphorylation interacts with many SH2-domain containing proteins and thereby enhances Arp2/3 complex formation during G-actin polymerization and cell migration^42^. It was further reported that the tyrosine phosphorylation of cortactin is essential for its role in receptor endocytosis, lamellipodium formation and cell migration^13^,^24^,^25^,^43^. In a recent study, we have shown that cortactin phosphorylation at S405 and S418 residues is required for its interaction with WAVE2 in facilitating G-actin polymerization and HASMC migration^9^. Although a large body of data demonstrates a role for cortactin in regulating cytoskeleton remodeling and cell migration, only a few studies show that cortactin also plays a role in the modulation of cell proliferation^26^,^27^,^28^. Since our previous study showed a role for cortactin in MCP1-induced HASMC migration, we asked the question whether cortactin has any role in MCP1-induced HASMC proliferation. In this context, it is interesting to note that depleting cortactin levels negates MCP1-induced HASMC proliferation and this effect correlates with blockade of p21Cip1 degradation. It was also reported that depletion of cortactin levels leads to upregulation of p21Cip1 levels at both mRNA and protein levels in FaDu cells^27^. In understanding the mechanisms of p21Cip1 downregulation by MCP1, initially we tested the role of cortactin deacetylation. Although inhibition of HDAC6 prevents MCP1-induced cortactin deacetylation, it had no effect on MCP1-induced p21Cip1 degradation, suggesting a lack of a role for cortactin deacetylation in MCP1-induced HASMC proliferation. Similarly, although cortactin phosphorylation at S405 and S418 residues is required for MCP1-induced HASMC migration, it appears that cortactin phosphorylation at these residues is not required for MCP1-induced HASMC proliferation, as S405A and S418A cortactin mutants did not affect MCP1-induced p21Cip1 degradation or HASMC DNA synthesis. Using mass spectrometry, some reports have identified Y421, Y446, Y453, Y470 and Y486 residues as potential sites of human cortactin tyrosine phosphorylation^13^,^44^. In addition, other studies have shown that phosphorylation of cortactin at Y421, Y470 and Y486 residues is needed for its role in cell migration^13^,^43^. However, the role of cortactin tyrosine phosphorylation in the modulation of cell proliferation is not clear. In this context, the present study shows that MCP1-induced p21Cip1 degradation and HASMC proliferation require phosphorylation of cortactin at Y446 but not Y421 residue. In addition, other reports showed that cortactin phosphorylation at Y446 residue was involved in the protection of HeLa cells from hyperosmotic stress-induced apoptosis^23^. A large body of data shows that p21Cip1 undergoes either ubiquitin-dependent or independent proteasome-mediated degradation in many cell types^45^,^46^. It was also reported that p21Cip1 translocation from the nucleus to the cytoplasm and its degradation require its Ser/Thr phosphorylation^47^,^48^. In the present study, we show that Y446F cortactin mutant prevents MCP1-induced p21Cip1 translocation from the nucleus to the cytoplasm, suggesting a role for cortactin tyrosine phosphorylation in MCP1-induced p21Cip1 nuclear export. It was reported that cortactin phosphorylation at Y466 residue promotes its deacetylation and prevents mouse fibroblast spreading^49^. However, in the present study we found that Y421F or Y446F cortactin mutants have no effect on MCP1-induced cortactin deacetylation or its serine phosphorylation. These findings infer that tyrosine phosphorylation of cortactin does not lead to its deacetylation or serine phosphorylation in MCP1-induced p21Cip1 nuclear export and its degradation. In other words, tyrosine phosphorylation of cortactin alone is sufficient in mediating MCP1-induced p21Cip1 nuclear export and its degradation.

It is noteworthy that besides MCP1, thrombin but not PDGF-BB stimulates cortactin phosphorylation at Y446 residue and this event is required for thrombin-induced p21Cip1 degradation and HASMC proliferation. Although, PDGF-BB induced cortactin phosphorylation at other tyrosine residues, those sites appear to be not required for PDGF-BB-induced p21Cip1 degradation or HASMC proliferation. Thus, these findings indicate that the involvement of cortactin phosphorylation at Y446 residue in p21Cip1 degradation is selective to GPCR agonists. Previous studies have reported that PDGF-BB induces tyrosine phosphorylation of murine cortactin on Y421, Y466 and Y482 residues in the mediation of lamellipodium formation and cell migration^13^,^16^,^43^. Based on these observations as well as the present findings that PDGF-BB induces cortactin phosphorylation at site(s) other than Y446 residue in HASMCs, a role for cortactin tyrosine phosphorylation in PDGF-BB-induced cell migration cannot be eliminated.

In elucidating the upstream mechanisms of cortactin tyrosine phosphorylation by MCP1, we found that MCP1 activates Fyn more robustly than the other SFK members and depleting its levels inhibits MCP1-induced cortactin tyrosine phosphorylation, p21Cip1 degradation and HASMC proliferation. Fyn has also been shown to co-localize with cortactin in the mediation of metastatic murine melanoma cell migration^50^. Initially, it was discovered that cortactin was a substrate for Src^35^, although the later studies have shown that several other non-receptor tyrosine kinases including ABL, FER and/or Syk could also mediate its tyrosine phosphorylation^16^,^21^,^22^,^51^. Interestingly, it was demonstrated that FER kinase phosphorylates cortactin downstream to Fyn in mediating osmotic stress-induced cytoskeletal reorganization and cell volume^52^. Based on this finding, it may be suggested that MCP1 may activate FER downstream to Fyn in mediating p21Cip1 degradation and HASMC proliferation, which needs further experimentation. Since a role for several non-receptor tyrosine kinases in cortactin phosphorylation has been reported, it is possible that various tyrosine kinases may be involved in the phosphorylation of cortactin at different tyrosine residues in response to different agonists in mediating cell migration or cell proliferation. Although activation of SFKs by GPCR agonists such as thrombin requires calcium^37^, the present results show that MCP1 induces cortactin tyrosine phosphorylation and p21Cip1 degradation via activation of Fyn in a manner that is independent of calcium mobilization. In our previous study, we have shown that MCP1 activates Gαq/11 via CCR2 in the modulation of HASMC migration^9^. Based on this observation, we suspected that Gq/11 might be involved in MCP1-induced p21Cip1 degradation and HASMC proliferation. However, siRNA-mediated depletion of Gαq/11 levels had no effect on MCP1-induced p21Cip1 degradation, suggesting a role for other G proteins in this effect. Indeed our results show that PT, a Gi/o-specific inhibitor, attenuates MCP1-induced Fyn and cortactin tyrosine phosphorylation leading to blockade of p21Cip1 degradation and HASMC proliferation. These findings are also consistent with the previous reports that Gi/o mediates MCP1-induced VSMC proliferation^53^. In addition, it was reported that thrombin-induced Src activity and fibroblast proliferation are PT-sensitive^54^. On the basis of these observations and present findings, we assume that Gi/o plays an important role in the modulation of Fyn activation leading to cortactin phosphorylation at Y446 residue, which in turn, promotes p21Cip1 translocation from the nucleus to the cytoplasm and its degradation. Many studies suggest that CCR2 is the major GPCR mediating MCP1-induced chemotactic and mitogenic signaling in various cell types^9^,^36^. It is also important to note that despite the presence of both CCR2 and CCR4 receptors in HASMCs, only CCR2 but not CCR4 is involved in MCP1-induced Fyn and cortactin tyrosine phosphorylation, p21Cip1 degradation and HASMC proliferation. Furthermore, although CCR2 couples to various G proteins, including Gq/11, G14/16 and Gi/o^13^,^55^,^56^, only CCR2 coupled to Gi/o proteins mediates MCP1-induced Fyn and cortactin tyrosine phosphorylation, p21Cip1 degradation and HASMC proliferation. The role of CCR2 in HASMC proliferation can also be supported by the findings that CCR2 mediates MCP1-induced VSMC proliferation during neointimal development^57^. In addition, other reports have shown that the Src family of tyrosine kinases are activated by MCP1 via CCR2 in monocytes and endothelial cells^58^. Furthermore, MCP1 signaling via CCR2 has been reported to enhance macrophage trafficking and immune responses in the pathogenesis of atherosclerosis^59^. On the basis of all of these observations and our present findings, we conclude that CCR2, upon activation by MCP1, triggers Gi/o-Fyn-dependent cortactin tyrosine phosphorylation, which in turn, facilitates p21Cip1 translocation from the nucleus to the cytoplasm and its degradation leading to HASMC proliferation.

**Figure 8:**
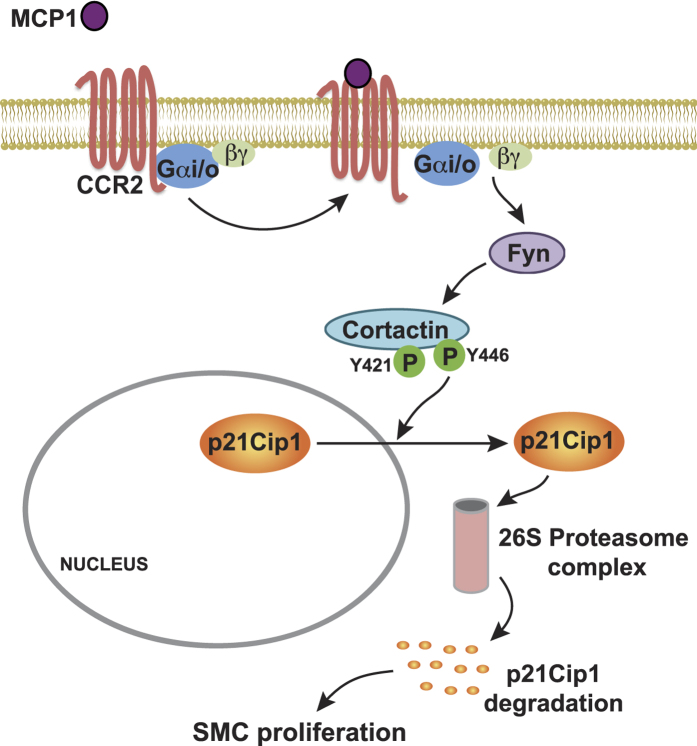
A schematic diagram showing the potential mechanism(s) of cortactin-mediated p21Cip1 nuclear export and degradation in facilitating MCP1-induced HASMC proliferation.

In summary, as shown in Fig. 8, cortactin phosphorylation at Y446 residue via CCR2-Gi/o-mediated Fyn activation plays an important role in the translocation of p21Cip1 from the nucleus to the cytoplasm and its proteasome-mediated degradation facilitating HASMC proliferation in response to GPCR but not RTK agonists.

## Materials and Methods

### Reagents

CCR2 antagonist (SC-202525), CCR4 antagonist (SC-221406) HDAC6 inhibitor (SC-223877), HDAC8 inhibitor PCI-34051 (SC-364566), anti-cortactin (SC-11408), anti-Gαq (SC-393), anti-Gα11 (SC-394; SC-390382), anti-GFP (SC-9996), anti-Fyn (SC-365913), anti-Lyn (SC-15), anti-p21Cip1 (SC-6246), anti-p27Kip1 (SC-528), anti-p57Kip2 (SC-1040), anti-pYes (SC-130182), anti-Yes (SC-8403) and anti-β-Tubulin (SC-9104) antibodies were obtained from Santa Cruz Biotechnology, Inc. (Santa Cruz, CA). Anti-pSrc (2101) antibodies were purchased from Cell Signaling Technologies (Beverly, MA). Anti-phosphoserine/threonine antibody (ab17464), anti-proteasome 20Sα + β (ab22673), and pFyn (ab192172) were bought from Abcam (Cambridge, MA). Anti-pLyn (04–375), anti-Src (05–184), anti-phosphotyrosine (05–777) antibodies were obtained from Millipore (Temecula, CA). Proteasome 20Sα1, 2, 3, 5, 6 & 7 subunits monoclonal antibody (BML-PW8195-0100) was obtained from Enzo Life Sciences (Farmingdale, NY). Goat anti-rabbit HRP (31460) and goat anti-mouse HRP (31437) antibodies, ambion silencer validated human Fyn siRNA (AM51331), BAPTA-AM (B6769), Lipofectamine 3000 transfection reagent (L3000-015), Hoechst 33342 (H3570), Prolong gold antifade mounting medium (P36930), Medium 231 (M231-500), smooth muscle growth supplements (S-007-25), and gentamicin/amphotericin solution (R-015-10) were obtained from Thermo Scientific (Waltham, MA). Protein A sepharose CL-4B (170780-01), Protein G sepharose Fast flow (17061801) and ECL Western blotting detection reagents (RPN2106) were received from GE Healthcare (Pittsburgh, PA). Human cortactin siRNA (ON-TARGETplus SMARTpool L-010508-00-0005) and control non-targeting siRNA (D-001810-10) were purchased from Dharmacon RNAi Technologies (Chicago, IL). Anti-CCR2 antibody (NB110-55674) was purchased from Novus Biologicals (Littleton, CO). Rhodamine phalloidin (00027) was bought from Biotium (Hayward, CA). [^3^H]-Thymidine (specific activity 20 Ci/mmol) was obtained from Perkin Elmer Life Sciences (Waltham, MA). Pertussis toxin from *Bordetella pertussis* was purchased from Sigma-Aldrich (St. Louis, MO). PP1 (567809), PP2 (529573) and PP3 (529574) were bought from Calbiochem (Billerica, MA).

### Cell culture

Human aortic smooth muscle cells (HASMCs) were purchased from Invitrogen, sub-cultured in medium 231 containing smooth muscle cell growth supplements and gentamicin/amphotericin. The cells were used between 4–10 passages for all the experiments.

### Plasmid constructs and site-directed mutagenesis

GFP-tagged human cortactin expression plasmid was a gift from Dr. Kenneth Yamada (National Institutes of Health, NIDCR) (Addgene plasmid # 50728). Myc-tagged human cortactin expression plasmid was a gift from Dr. Scott Weed (West Virginia University). The Y421F, Y446F, Y453F, Y470F and Y486F mutations in cortactin were inserted by QuickChange Lightening Site-directed mutagenesis kit (Agilent Technologies; Catalog No. 210518) using GFP-tagged or Myc-tagged cortactin expression vector and the following primers: Y421F mutant: forward, 5′-CCCTCGAGCCCCGTCT**T**TGAGGATGCG-3′, reverse, 5′-CGCATCCTCA**A**AGACGGGGCTCGAGGG-3′; Y446F mutant:forward, 5′-CGGAGCCCGTGT**T**CAGCATGGAGGC-3′, reverse, 5′-GCCTCCATGCTG**A**ACACGGGCTCCG-3′; Y453F mutant:forward, 5′-CATGGAGGCCGCTGACT**T**CCGAGAGGC-3′, reverse, 5′-GCCTCTCGG**A**AGTCAGCGGCCTCCATG-3′; Y470F mutant:forward, 5′-CCTATGCCACAGAGGCTGTCT**T**TGAAAGCGCAG-3′, reverse, 5′-CTGCGCTTTCA**A**AGACAGCCTCTGTGGCATAGG-3′; Y486F mutant: forward, 5′-GCAGAGGACAGCACCT**T**CGATGAGTACGAG-3′, reverse, 5′-CTCGTACTCATCG**A**AGGTGCTGTCCTCTGC-3′. All the above five tyrosine residues in cortactin were mutated to phenylalanine (Y5F) by QuickChange Lightening Multi site-directed mutagenesis kit (Agilent Technologies; Catalog No. 210515) using GFP-tagged cortactin expression vector and all the above-listed primers in the same reaction. The mutant nucleotides are shown in bold letters and the constructs with mutations were confirmed by DNA sequencing. Plasmid DNAs were purified using EndoFree Plasmid Maxi Kit (Catalog No. 12362; Qiagen) and used in the transfection experiments.

### Coimmunoprecipitation

After rinsing with cold PBS, cells were lysed in 400 μl of lysis buffer (PBS, 1% Nonidet P40, 0.5% sodium deoxycholate, 0.1% SDS, 100 μg/ml PMSF, 100 μg/ml aprotinin, 1 μg/ml leupeptin, and 1 mm sodium orthovanadate) for 20 min on ice. The cell extracts were cleared by centrifugation at 14,000 rpm for 20 min at 4 °C. The cleared cell extracts containing an equal amount of protein from control and the indicated treatments were incubated with the appropriate antibodies overnight at 4 °C, followed by incubation with protein A/G-Sepharose CL4B beads for 3 hrs with gentle rocking. The beads were collected by centrifugation at 1000 rpm for 2 min at 4 °C and washed four times with lysis buffer and once with PBS. The immunocomplexes were released by heating the beads in 40 μl of Laemmli sample buffer and analyzed by Western blotting for the indicated molecules using their specific antibodies.

### DNA synthesis

DNA synthesis was measured by [^3^H]-thymidine incorporation as described previously and expressed as counts/min/dish^60^.

### Immunofluorescence staining

After appropriate treatments, HASMC were fixed with 3.7% paraformaldehyde for 15 min, permeabilized in 0.3% Triton-X-100 for 15 min, blocked with 3% BSA for 1 hr and incubated with mouse anti-p21Cip1 (1:100) and rabbit anti-proteasome 20Sα + β (1:500) antibodies followed by incubation with Alexa Fluor 488-conjugated goat anti-mouse and Alexa Fluor 568-conjugated goat anti-rabbit secondary antibodies (1:500), respectively. After washing with PBS, cells were counterstained with Hoechst 33342 (1:2000) and mounted onto glass slides with Prolong Gold antifade mounting medium. Fluorescence images of cells were captured using an inverted Zeiss fluorescence microscope (AxioObserver Z1) with 40X/NA 0.6 objective and AxioCam MRm camera without any enhancements using the microscope operating and image analysis software AxioVision verstion 4.7.2 (Carl Zeiss Imaging Solutions GmbH).

### Preparation of cytoplasmic and nuclear fractions

After appropriate treatments the cytoplasmic and nuclear fractions of HASMCs were prepared using NE-PER nuclear and cytolasmic extraction reagents as described in the manufacturer’s (Catalog No. 78833, Thermo Scientific) instructions.

### Western Blotting

An equal amount of protein from control and treatment samples was separated by SDS-PAGE and analyzed by Western blotting for the indicated molecules using their specific antibodies as described previously with minor modifications^9^. Briefly, all the primary antibodies were used at 1:500 to 1:1000 dilutions and incubated overnight at 4 °C. Goat anti-rabbit or goat anti-mouse antibodies conjugated with HRP were used at 1:4000 to 1:5000 dilutions and incubated for 2 hrs at room temperature. Blots were developed using ECL detection reagents and the blots were quantified by densitometry using ImageJ software (National Institutes of Health).

### Transfections

HASMCs were transfected with non-targeted control or on-target siRNA at a final concentration of 100 nM using Lipofectamine 3000 transfection reagent according to the manufacturer’s instructions. Cells were transfected with plasmid DNAs at a final concentration of 2.5 μg/60 mm dish or 5 μg/100 mm dish using Lipofectamine 3000 transfection reagent according to the manufacturer’s instructions. After transfections, cells were growth-arrested for 36 hrs and used as required.

### Statistics

All the experiments were repeated three times, and the data are presented as Mean ± S.D. One-way ANOVA was used to perform statistical analysis and the p values < 0.05 were considered statistically significant.

## Additional Information

**How to cite this article**: Janjanam, J. and Rao, G. N. Novel role of cortactin in G protein-coupled receptor agonist-induced nuclear export and degradation of p21Cip1. *Sci. Rep*. **6**, 28687; doi: 10.1038/srep28687 (2016).

## Supplementary Material

Supplementary Information
