# Supplementary material for: Novel role of cortactin in G protein-coupled receptor agonist-induced nuclear export and degradation of p21Cip1
Source: Sci Rep. 2016 Jul 1;6:28687. doi: 10.1038/srep28687 (PMC4929470; doi:10.1038/srep28687)
Supplement: Supplementary Information [file srep28687-s1.pdf]

Novel role of cortactin in G protein-coupled receptor agonist-induced nuclear export and degradation of p21Cip1

Jagadeesh Janjanam and Gadiparthi N. Rao

Department of Physiology, University of Tennessee Health Science Center,
Memphis, TN 38163, USA

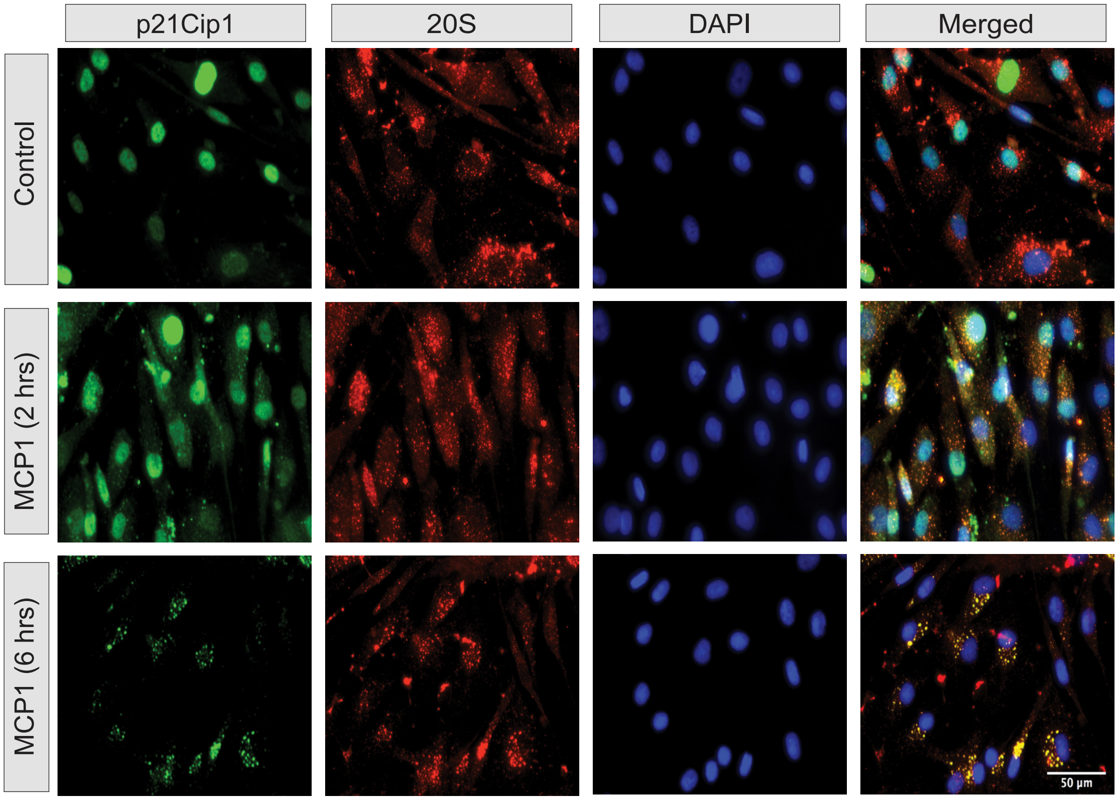

Figure S1. MCP1 induces p21Cip1 co-localization with 20S proteasomes. Growth-arrested HASMCs were treated with vehicle or MCP1 (50 ng/ml) for 2 hrs or 6 hrs and co-immunostained for p21Cip1 and proteasome 20Sα/β subunits using their specific antibodies.

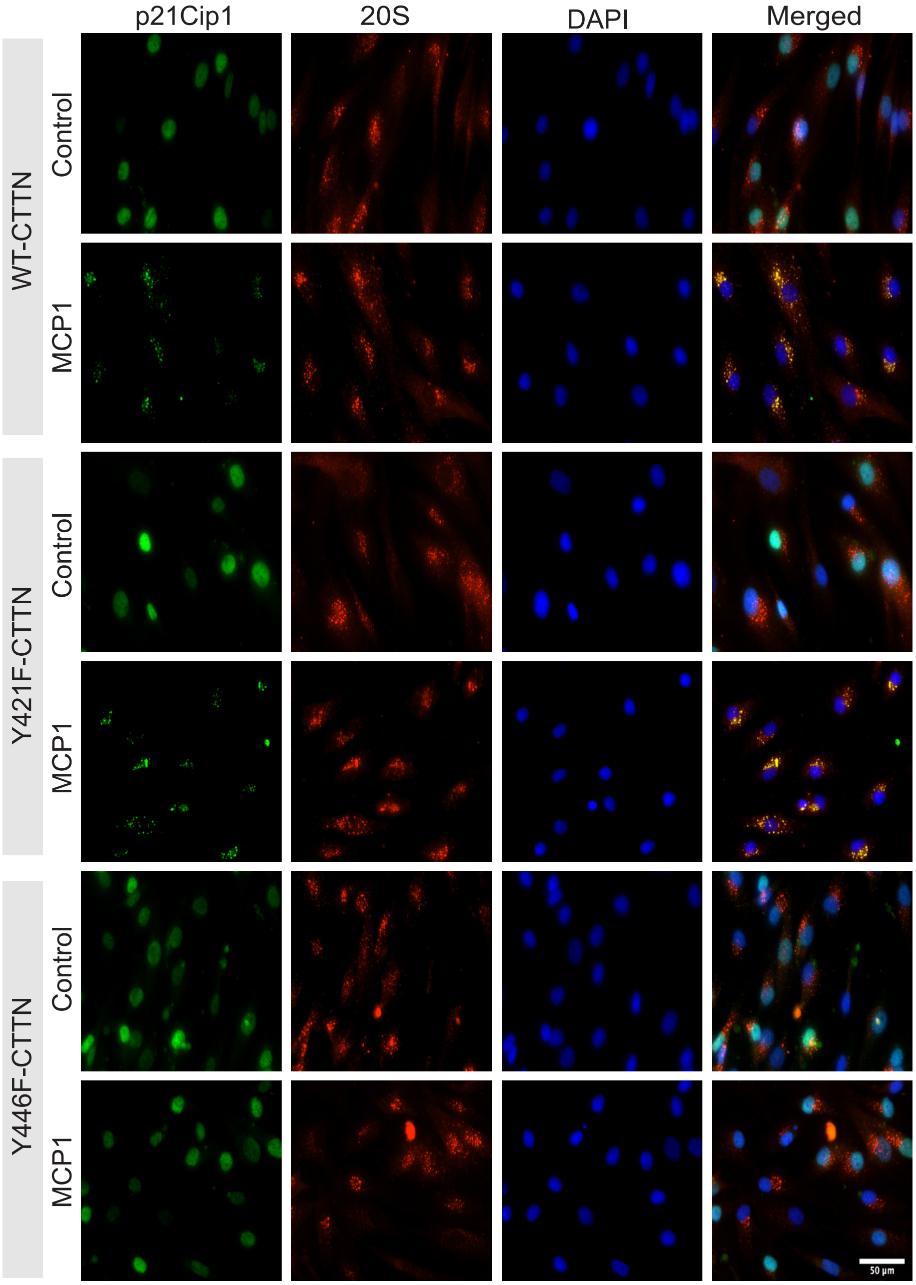

Figure S2. Cortactin phosphorylation at Y446 residue is required for MCP1-induced p21Cip1 nuclear export and its proteasomal degradation. HASMCs were transfected with Myc-tagged WT or mutant (Y421F or Y446F) cortactin expression vectors, growth-arrested, treated with vehicle or MCP1 (50 ng/ml) for 6 hrs and co-immunostained for p21Cip1 and proteasome 20Sα subunit using their specific antibodies.

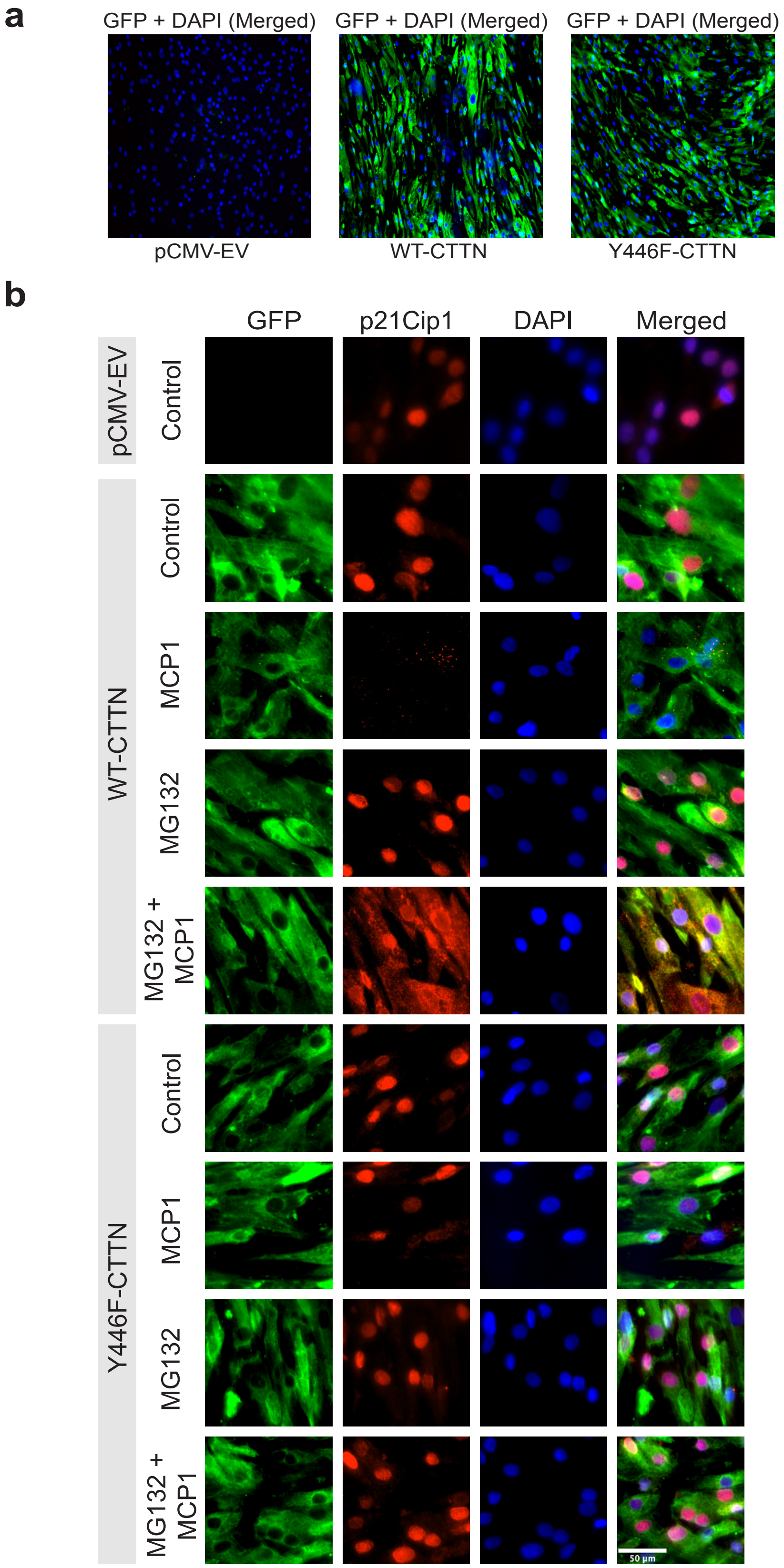

Figure S3. Cortactin phosphorylation at Y446 residue is required for MCP1-induced p21Cip1 nuclear export and degradation. A. Cells were transfected with untagged pCMV empty vector (pCMV-EV), GFP-tagged WT or Y446F mutant cortactin expression vectors and 2 days later cells were stained with DAPI. Cells were visualized under Zeiss Fluorescence microscope (AxioObserver Z1) at 10X magnification using DAPI and GFP filters for the nucleus and cortactin, respectively. B. Cells that were trasnfected with pCMV-EV, GFP-tagged WT or Y446F mutant cortactin expression vectors and growth-arrested were treated with vehicle or MCP1 in presence and absence of MG132 for 6 hrs and immunostained for p21Cip1 using its specific antibody. The cells were visualized under GFP, RFP and DAPI filters for cortactin, p21Cip1 and nucleus, respectively.

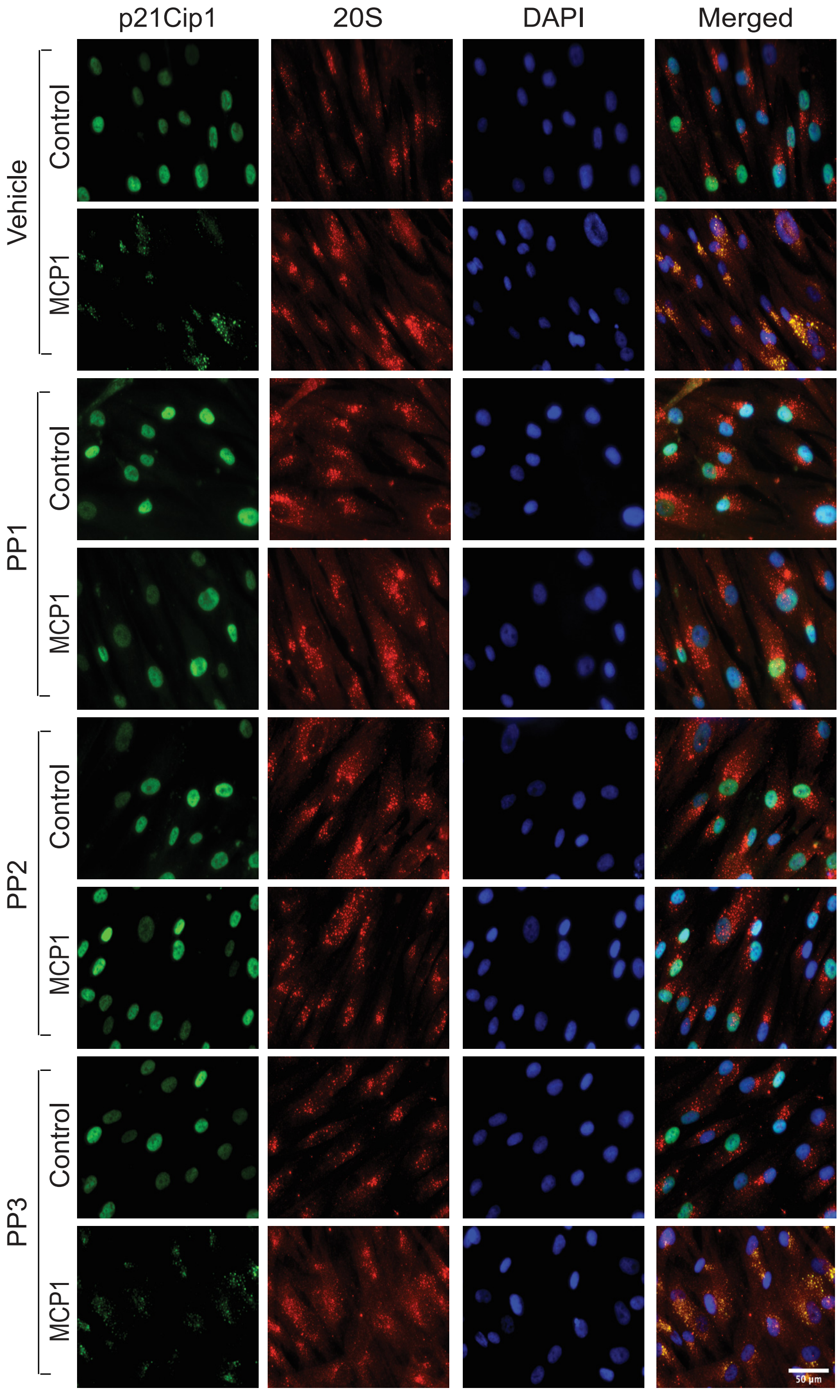

Figure S4. Inhibition of SFKs blocks MCP1-induced p21Cip1 nuclear export and its co-localization with 20S proteasomes. Growth-arrested HASMCs were treated with vehicle or MCP1 (50 ng/ml) in the presence and absence of PP1 (10 μM), PP2 (10 μM), or PP3 (10 μM) for 6 hrs and co-immunostained for p21Cip1 and proteasome 20Sα/β subunits using their specific antibodies.
